# Critical Thinking for Writing Using Facebook Under COVID-19 Lockdown: A Course Model for English Literature Students

**DOI:** 10.3389/fpsyg.2022.903452

**Published:** 2022-06-03

**Authors:** Elaf Almansour, Mustafa Kurt

**Affiliations:** Department of English Language Teaching (ELT), Near East University, Nicosia, Cyprus

**Keywords:** English language, writing skill, literature, critical thinking, Facebook, COVID-19 pandemic

## Abstract

The aim of this paper is to explore the effectiveness of critical thinking for improving the writing skill of undergraduate Arab students who study English Literature at Saudi universities under lockdown circumstances due to the COVID-19 pandemic. At the same time, it explores the impact of implementing Facebook as an online Constructivist tool to improve this skill. A general overview of the status of English language education in Saudi Arabia is briefly presented to shed light on the ongoing English language challenges in learning writing for undergraduate students in the English language and literature departments, which got more manifested due to the current status of education mode with the emergence of the pandemic. Two-group posttest-only randomized experiment was employed to evaluate the effectiveness of the proposed model, using the infusion and constructivism approaches. A total of 40 students enrolled in a literature course at a private university in Saudi Arabia participated in the experiment. The treatment was conducted through utilizing Facebook. The results demonstrated that students’ improvement in English writing was due to the combination of the infusion of a set of critical skills and the constructivist teaching and learning mode.

## Introduction

Various studies and research have been conducted with exploring challenges facing English language and literature students in Saudi universities. Among the various conclusions, following need a mention: limited time of English language courses, intensive load of curricula, teaching methodologies, lack of the target language environment, lack of adequate up-to-date effective instructions in the classroom, and learners’ lack of motivation, to name some (Hammami, 2002; Rababah, 2005; Ansari, 2012; Al Hosni, 2014; Hussein and Elttayef, 2016; Omer, 2018). Hammami (2002) elaborates that the core problem of Arab learners of English lies in the pre-packaged language teaching curricula that are imported for the students but are not based on their needs; these pre-packaged curricula are delivered in a tedious manner that slackens students’ improvement. Alrabai (2017) adds that English teachers in Saudi Arabia are always equipped with an identical fixed syllabus with guidelines and deadlines that they are required to apply and follow, and this strongly prescriptive nature of the curriculum likely reinforces student dependency on the teacher. Omer (2018) sheds light on the psychological factors that hinder students from learning English, such as the fear of making mistakes, anxiety, shyness, lack of confidence, and lack of motivation. These factors result in weaknesses in students’ English language level which consequently affects their academic attainment in other courses and their attitude.

Saying this, once COVID-19 has been unanimously considered a global pandemic, the previously mentioned challenges became more complex to cope with due to the closure of universities and a switch over to online education in general and virtual learning in particular. Universities in Saudi Arabia start to use virtual platforms as the main tool for delivering learning content and facilitating communication between education stakeholders, teachers, and students. However, immediate impacts have been witnessed as “the pandemic adds a further degree of complexity to higher education” (Pedro et al., 2020, p. 11), and many prior unresolved issues surface in a more visible manner. In addition, new challenges arise that can be categorized as the following: stakeholders’ readiness, mainly teachers, and students to adapt to online teaching, online platforms and resources compatibility, and curriculum modification to provide (at least) same outcomes as in class teaching.

Nevertheless, this pandemic still offered the opportunity to reconsider the status of English language and literature teaching in non-English speaking countries, such as Saudi Arabia and reconsider and redesign the teaching and learning strategies and syllabus, i.e., rethinking about the skills students acquire in their education and whether these skills offer them authentic learning outcomes and sufficiently prepare them for working life. Therefore, this course adjusts new teaching approaches, constructivism, and infusion, to propose a new learning model for English language and literature undergraduate students that aim to enhance their critical thinking, better develop their language skills, and reinforce their learning experience. Besides, it aims to encourage students to be involved and interact using an innovative and interactive studying platform that can enhance and develop new skills to learning content, facilitates constructivist learning, and most importantly individualized monitoring and feedback to ensure that no student is left behind.

Laderman (2020) says that due to the crisis of COVID-19, teachers and students both find themselves compelled to embrace the digital academic experience as the ultimate option for continuing the teaching-learning process. Ekoc (2014) claims that social media would better engage students in the learning process as active learners, team builders, collaborators, and discoverers. According to the report of Jones and Fox (2009), among the various types of social media, Facebook is highly used by students, as 85–99 percent of all students use this platform. This is attributed to the following: Facebook does not require training due to its popularity, so students would not feel frustrated toward preparing and using a new learning mode. Supporting this, several studies (Li and Pitts, 2009; Mazer et al., 2009; Park et al., 2009; Munoz and Towner, 2010; Lewis and Nichols, 2012) have concluded that generally students had positive attitudes toward using Facebook in the classroom claiming that it is very user-friendly platform that does not require any special skills or settings. Thus, it is considered as a convenient online learning platform for the teachers and students.

In addition, Facebook can develop a constructivist learner centered online learning, intellectual participations, and discussions, and hence can illuminate critical thinking learning experience more constructively, as it has the potential to motivate student inquiry and create a context in which they learn cooperatively and collaboratively, promoting both reflection and critical thinking (Black, 2005).

Thus, implementing Facebook in this study aims at achieving two main objectives (1) to improve English writing skills and (2) to encourage critical thinking in the context of online discussions, as student-to-student interaction create freer discussion and analysis of ideas (Seo, 2007) as students can freely express themselves to their peers in more comfortable zone away from classroom restrictions; As well as it exposes students to other peers with different thoughts and ideas than their own and grants them the chance to respond to these differences; hence, they learn from each other by interacting with and evaluating others’ ideas. Besides, the role of the instructor in monitoring facilitating, interpreting, and synthesizing of information and ideas (Wang, 2009) is also essential. For instance, it is mainly the instructor’s responsibility to emphasis avoiding any personal bias in these discussions and comments.

### Aim of the Study

This study attempts to show that significant changes do not necessarily require a giant reform; small changes would return with great results. By adopting two well-known approaches, the constructivist and infusion approaches for the sake of achieving deeper and life-long learning experience, the current study aims to redesign the teaching instruction by infusing Paul and Elder’s critical thinking skills into a literature course in an Arab context, an attempt to use the current curriculum for improving both students’ critical thinking and writing using Facebook under COVID-19 lockdown.

### Research Questions

The current study attempts to answer the following questions:

1. To what extent can critical thinking improve students’ writing?
2. To what extent can Facebook as a learning platform stimulate students’ writing constructively?
3. What is students’ perception of this critical thinking-infused constructivist course model?

### Literature Review

#### Status of English in Saudi Arabia

English language is taught in Saudi Arabian as a foreign language. It is not widely used nor practiced in everyday life, and the intensity of the English curricula is generally low but varies according to the studying level. Students join universities with low to intermediate level of English, which does not qualify them to study their fields in English, as most of the university courses are taught in English. To fill this language gap, universities offer foundation courses in English in which students complete five levels of pure English before they start their specializing courses. However, these courses fail to fully bridge this gap due to numerous reasons, the most important of which is the methodology of teaching English. In fact, English is taught theoretically similarly to mathematical formulas, i.e., students memorize new words and grammar rules by heart with no practical use.

Reform in English curriculum, teaching methods, teaching aids, and teaching atmosphere and students’ attitudes have been called for several years ago (Gordon, 1980; Al Ahaydib, 1986) but could not find its way till the recent time. In response to the *2030 vision* of Saudi Arabia, serious and productive actions start to take place in all fields of education especially English language teaching due to the awareness of Saudi Arabia Government of its reformative role in developing the country and coping with the dramatic and accelerating changes happening worldwide. Educational leaders and stakeholders have started to work on developing new modern curricula that facilitate progress and have already published a sophisticated range of education outcomes for higher education to level with the requirements of the job market and to prepare well equipped, highly educated generations (Full text of Saudi Arabia’s Vision 2030, 2016). Their goals include refining current curricula, implementing technology, and training teachers to use effective teaching methods and best practices, to name just a few. Such reform steps must consider that with the massive spread of information and accelerating advancement of technology, students’ academic achievement should not be measured by the amount of information they store in their brains, but the way they deal with it, filter it, make use of it, and connect it with their real-life experiences. The focus in teaching and learning must shift from “what to learn” to “how to learn” in an interactive and engaging manner.

#### Critical Thinking and Its Importance for Better Learning

Critical thinking is commonly known as sets of skills applicable in all fields of study that lead to better learning. However, it is hard to agree on one unified definition for it in educational programs and courses as each definition has its own core elements and standards, as Halanon (1995) stated “no single definition of critical thinking is widely accepted” (p. 75). Therefore, some definitions can be more appropriate for certain programs or courses than others. For instance, for English literature courses, theories founded by Glaser (1941); Lipman (2003), or Paul and Eldar (2007) may be more adequate, as critical thinking is referred to as a skillful thinking process, in which the skills of language are used as tools to examine and analyze the content. Thus the skills of thinking, reading, and writing critically are intimately interrelated; students are supposed to use clear and accurate language in interpreting learning content, appraising evidence, evaluating arguments, and drawing warranted conclusions that can be tested, a process that enables students to reconstruct their knowledge of English literature by reaching accurate judgments based on wider experiences (Glaser, 1941). They would also learn how to detect vagueness and ambiguity from clarity and precision within the learning content they study in order to articulate clear, relevant, and significant thoughts and points (Paul and Eldar, 2007).

However, critical thinking is hardly practiced by undergraduate students of English language and literature; and if that happens, it is limited to sets of analysis, evaluation, or reader-response questions which have no criteria to be met. Students’ answers are neither organized nor assessed as the students are not trained to answer critical thinking questions, they fail to use clear and accurate language to represent their ideas, and their answers lack common critical thinking standards, such as depth, breadth, and significance. Hence, lecturers or educators usually notice that students forget the learning content soon after the end of the courses and rarely connect the acquired knowledge to other courses. Such obstacles can be attributed to the unavailability of the academic courses in English Language and Literature departments that aim to foster critical thinking.

On these grounds, Paul and Elder (2019) have proposed a model of critical thinking, which consists of a set of reasoning skills that examine the content sensitively, a group of intellectual standards that aims to self-assessing and correcting, and intellectual traits that differentiate to what extent thinkers are capable of being fair-minded and critical. Students cannot engage in better thinking unless they learn how to employ criteria and standards by means of which they can assess their thinking for themselves (Lipman, 2003). Paul and Elder (2019) also assure that the four language skills are interrelated with critical thinking and need to be practiced together. Based on their theory, students can improve their thinking and language skills from spontaneous and superficial levels to deep and long-life learning through persistent practice of critical thinking skills and constant assessment in language.

#### The Infusion Approach for Teaching Critical Thinking

The infusion approach is based on teaching students critical thinking skills by using content and context in which they can explicitly use these skills (Ennis, 1989). Weinstein (1995) argues that critical thinking should be embedded in other subjects because “whatever the dispositions, skills, and strategies used, they need to be identified, contextualized, and exercised within the regular curriculum if critical thinking is to take a secure place in teaching and learning” (p. 40). Thus, the infusion approach emphasizes the process of acquisition of thinking skills through the context of content learning and instruction and instils critical thinking skills along with the study subjects (Swartz, 1992). Furthermore, applying the infusion approach can be one of the best options in reforming English literature curriculum on the short term as it does not require a complete change of the materials, but to redesign the course instruction keeping critical thinking in mind, and shifting the teaching from teacher-centered to student-centered. These can be simple efforts but can make a big difference.

Paul and Elder’s critical theory is infused in the current study in a literature course to foster undergraduate students’ critical thinking and improve their writing skill. It is selected because of its universality and applicability to language and literature courses, as (Paul and Elder, 2019, p. 128) claim that its elements and standards are “present in all reasoning of all subjects in all cultures for all time.” They also add that mastering this theory in one context enables students to apply it in other contexts, whether academic studies or real-life experiences; hence mastering it would also contribute to improving students’ content learning in future courses. Paul and Elder (2019) believe that students have to consistently learn how to write as they write to learn. They define substantive writing as “the ability to identify important ideas and express significant implications of those ideas in clear and precise writing” (p. 10). Paul and Elder’s framework for writing critically, which is built on a set of reasoning elements and of intellectual standards for assessing the elements, requires students to be able to apply one or more of the following five levels: (1) paraphrasing accurately the learning content into their own and identifying the essential meaning of it; (2) explicating the thesis statement of the learning content, by stating, elaborating, exemplifying, and illustrating the thesis of each part or paragraph; (3) analyzing their thinking for assessing it by identifying; (4) evaluating and assessing the text using the intellectual standards: clarity, precision, accuracy, relevance, significance, depth, breadth, logic, and fairness; and finally (5) practicing role-playing the thinking of the author to demonstrate their critical thinking analysis of the text. On this basis, it is crucial for students to understand the intimate relationship between thinking and writing, any significant deficiency in thinking entails a parallel deficiency in writing and vice versa (Paul and Elder, 2019).

#### Facebook as a Constructivist Tool for Online Learning

Constructivism is a well-researched theory in education which emphasizes that learning should be an active process through which students improve their learning skills and building knowledge within a supportive community (Taylor, 2009). One of the essential criteria of constructivism is the interaction among students during the learning process. Students use their prior knowledge and experiences for constructing new knowledge (Hoover, 1996; Driscoll, 2005) and share their ideas and experiences with their peers (Almala, 2006; Lim and Ismail, 2010; Alhojailan, 2012; LaRue, 2012). The role of educators or teachers shifts from the “sage on the stage [to] the guide on the side” (King, 1993, p. 30). Their role becomes more that of a counselor, consultant, and friendly critic (Brooks and Brooks, 1999); they prepare activities for the learning content, observe students, and provide assistance that keeps the learning process smoothly moving along.

The constructivist Carwille (2007), among others, states that it is crucial to apply the constructivist approach in online teaching because it encourages students to be active and motivated. With the massive advancement in technology, various Learning Management Systems (LMSs) are founded and implemented for educational purposes, whether formal such as Moodle or informal such as social media. As Weller et al. (2005) contend digital learning can contribute to improving the learning process in an innovative manner in which students generate and share subjective rather than objective experiences and thoughts to construct new knowledge (Von Glasersfeld, 1990). Social media applications, such as Facebook, Instagram, and Twitter, can help in changing the teaching mode from an instructional mode based on lecturing to a collaborative mode, in which students are engaged in discussions and exchange of ideas with their peers in an active and interactive process (Fosnot and Perry, 1996; Goktalay and Ozdilek, 2010). Each student becomes part of a community that works together to improve the learning content (Collins, 2009). Literature is rich with studies and researches that particularly investigate the effectiveness of social media as constructivist teaching tools. Lantz et al. (2013) found that social media can offer a collaborative language-learning space in which students combine learning content and communicative use of language if social media is integrated properly and its purpose clearly understood by students and educators (Kear, 2004).

Several studies have explored the impact of Facebook on education, considering several aspects, such as utilizing it in teaching and learning, stakeholders’ attitudes and perspectives, including teachers, students, and administrators, toward utilizing it for educational purposes and as an educational resource (Kabilan et al., 2010; Aydin, 2012) and the relationship between Facebook utilization and students’ motivation and engagement (Hyland, 2004; Junco, 2012). Furthermore, Facebook features facilitate communication without violating users’ privacy, as these groups do not necessitate their members to be mutual friends. In one study, Alshehri and Lally (2019) investigate university students’ attitude toward using social media in education at a Saudi university. Although the study finds that all the participants were familiar with social media and frequently used various applications and exhibited a positive attitude toward them, it indicates that social media are still not widely implemented in the education field in Saudi Arabia.

#### Facebook as a Learning Tool During COVID-19 Lockdown

During the COVID-19 pandemic, the abrupt closure of institutions and universities in Saudi Arabia has caused students to have significant challenges continue their academic attainment; hence, an immediate action was needed, that was basically implementing technology in education, developing online learning communities, and improving their capability to be more innovative in using the various applications to involve their students in a significant and accessible online teaching-learning experience, as well as cater their needs.

Social media, such as Facebook, Telegram, WhatsApp, and other has become inevitable for the continuity of education, namely teaching and learning the English language skills (Kerres, 2020; Wang et al., 2020). Selecting one application would be mainly based on how it can be used to facilitate constructive and effective learning, such as planning the lessons, posting the learning materials, and students-students and students-instructor’s interaction, with keeping students’ confidence and independent learning in mind.

Supporting studies of Low and Warawudhi (2016); Faryadi (2017), and Alhumaid et al. (2020), in which they refers to the significance of social media, mainly Facebook, in enhancing students’ English writing skill in an encouraging learning environment, as they found that students can improve their confidence, contentment, motivation, and perceptions about learning English. In addition, they conclude that Facebook can improve English learning and interaction between instructors and students, especially in big classes with variant ability students. Notwithstanding above, Facebook is selected for this study.

## Materials and Methods

A triangulation mixed methods design was used in the study and it is a type of design in which different but complementary data were collected on the same topic (Creswell et al., 2003). In this study, quantitative instruments were used to test the overall perception of the participants and their critical thinking and language skills improvement. Concurrent with this data collection, qualitative phase included the intervention that is designed and conducted by the researcher, a PhD candidate specializing in infusing critical thinking in English language and literature education, with 8-year experience as an EFL instructor. She successfully completed 40 training hour course: “How to Infuse Critical Thinking into Instruction” with The Foundation of Critical Thinking prior to this study. The reason for collecting both quantitative and qualitative data is to bring together the strengths of both forms of research to compare results and validate results.

### Research Instruments

Following are different but complementary tools that were used to answer the research questions, their results are triangulated for more reliable results:

#### Post-test

The post test is aimed to measure the improvement in students’ writing in both groups after completing the course and to see whether the skills of critical thinking impacted the performance of the experimental group. The posttest was assessed in terms of students’ language use and critical analysis: The language skills were assessed by meeting three essential criteria: (1) correct essay structure, (2) free of grammar mistakes and use a wide range of relevant terminology, and (3) correct in-text citation and referencing. In addition, a rubric set by Paul was adopted to measure students’ reasoning skills in terms of clarity, accuracy, precision, and well exemplification. Students’ performance was scored in both the reasoning and language rubrics according to a 10-point scale: 0–2 points for unacceptable (unskilled) writing; 3–4 points for poor (minimally skilled) writing; 5–6 points for mixed level writing (beginning skills); 7–8 points for commendable writing (skilled); and 9–10 points for fully meeting the criterion that is excellent and highly skilled writing (Appendix A). A *t* test is then done to determine if there is a significant difference between the writing level of the control and experimental groups.

##### Field Notes

Regular, detailed, and precise field notes were taken from both groups during the online teaching and learning to check the effectiveness of the intervention in achieving its purpose. The researcher observed and recorded students’ interaction, progress and involvement in each, and consequently to what extent each platform succeeded to stimulate students’ writing. The fields notes were used tobolster the results of other tools.

#### Questionnaire

A questionnaire of five sub-sections designed by the researcher aims to answer the third research question by finding out the experimental group students’ perception and attitude toward the intervention. A five-point Likert scale was used for 44 items questionnaire in terms of their overall perception of the intervention, their critical thinking and language skills improvement, and their attitude of implementing Facebook for learning and the instructor’s constructivist teaching style by indicating whether they *strongly agree*, *agree*, *disagree*, *strongly disagree*, or are *neutral* about the questionnaire items (Appendix B). In order to check the validity of the questionnaire, a group of PhD holders were asked to evaluate the questionnaire items and provide their comments and suggestions and a final draft was written accordingly; then Cronbach alpha was performed in order to check its reliability and the overall reliability ranged from (0.92) to (0.97), which revealed good reliability.

### Research Design

An only-posttest two-group randomized experimental design was used in this study. Qualitative and quantitative data were collected for more reliable and validated results. The intervention is designed and conducted by the researcher, a PhD candidate specializing in infusing critical thinking in English language and literature education, with 8-year experience as an EFL instructor. She successfully completed 40 training hour course: “How to Infuse Critical Thinking into Instruction” with *The Foundation of Critical Thinking* prior to this study.

The intervention took place during exceptional circumstances due to COVID-19 lockdown, when teaching the university courses was transferred to the online mode. Out of 16 weeks (one academic semester), all the students had already completed 6 weeks of study on campus, in which they were theoretically introduced to the main themes of the course. Two weeks were allocated for the mid and final exams; however, these exams are not used as tools as they included materials which are not included in this study. Thus, 8 weeks were assigned for online study, in which students had to do critical reading and analysis of two short stories. In this vein, both the experimental and control groups were taught by the same researcher *via* online mode with the former as per the new design of the course, and the latter as per the traditional way of lecturing at the university.

Unlike studies such as DiVall and Kirwin (2012) in which the researchers compared the pros and cons of the formal (Moodle) and informal (social media) online platforms, this study implemented both modes of teaching for other reasons. Moodle was used for the control group according to the university regulations, similar to other running courses, while the informal mode, namely Facebook, was selected by the researcher due to students’ familiarity with it and all its facilities.

#### Instruction Procedure for the Control Group

The researcher assumed the role of a lecturer with the control group lecturing them as in class using the university LMS. Prior to the lockdown, the first online lecture was devoted to introducing the LMS and how to use it during and after the lectures, as some students were unfamiliar with it. They were guided on how to download the presentations, to contact the lecturer *via* email, and to participate during the lecture; however, the only way of participation available for all the courses was the written chat. As the mode of teaching is lecturing, students do not interrupt lecturers during their presentations. However, unlike class lectures, attendance was not checked as making sure to involve all students was merely impossible.

#### Instruction Procedure for the Experimental Group

The experimental group resumed the course but with a constructivist teaching style using Facebook. A *Social Learning* group with its features, such as creating units and tracking members, progress for facilitating the teaching process was created and implemented. The learning content was organized in units that match the pacing schedule of the main course: there were three units, the introduction and two others for the learning content. In the first unit, different materials, such as infographics, graphs, and videos, were uploaded to introduce students to the concept of critical thinking in an attractive manner. Two more units were created for the learning content; each unit was allocated 3 weeks of study in order for the experimental group to be on the same pace as the control group.

In the first week on Facebook, the experimental group had a 2-h live meeting with the first hour devoted to introducing the theory and skills of critical thinking and the last hour allocated for discussion. In order to better familiarize the students with the critical thinking skills, they were informed to check the introductory unit on Facebook group and were provided with two short essays to read and think about prior to the meeting. Thus, the second-hour discussion was meant to be a warming up activity in which the instructor explicitly guided students to answer the set of critical thinking questions and encouraged them to share their thoughts.

After the students’ attempts to answer each question, the instructor explained the meaning of the keywords that students need to understand in order to answer the questions. For example, in the first question, students had to know that a thesis statement of a text is its key idea; it is essential to identify it in any text they read in order to clearly understand it and connect its meanings to other concepts from prior knowledge and experiences (Paul and Elder, 2019), and so on. By the end of the discussion, the students were provided with the answer-key to the questions, written by an expert in critical thinking, to better understand the procedure of applying the theory. By the end of the first meeting students were requested to review all the content posted in the introductory unit to reinforce their understanding and to post any questions about any vague points to be clarified and assessed by the instructor.

From the second week on, the students started to have only 1-h live meeting, in which some of them were asked to summarize in their own words what they had read, paraphrase it, and give examples on it from their experiences (Paul and Elder, 2019), while the others had to comment on their summaries and examples by applying the reasoning standards of clarity, accuracy, precision, relevance, depth, breadth, and significance (Paul and Elder, 2019). The second hour was up to the students to complete the above mentionedcritical thinking questions posted in the group at their own pace, as these questions were estimated to require approximately 1 h of work.

The students were to interact and collaborate on the Facebook group by posting their answers and assessing their peers’ answers using the *reply* and *tag* facilities for commenting, agreeing, disagreeing, and asking for clarification and explanation. In addition to learning the content, students had the chance to receive feedback on their language use and techniques. The researcher monitored students’ interaction and progression; she followed up their responses, probed their thoughts, and commented on them and highlighted their language mistakes by providing individualized feedback on each comment. Finally, the researcher made use of *Create Group Event* facility for posting details about the course pacing schedule such as the page count to read each week and the deadline for posting answers; hence, students received regular notifications regarding the course progression.

### Participants

Participants in this study are 40 English literature undergraduate students, enrolled in a third-year course at a private university in Saudi Arabia. The participants’ level in English is pre- to intermediate as they have completed five courses in general English in the foundation year plus a course in academic writing. They are supposed to be well-equipped to write well-organized and coherent paragraphs and essays. Prior to the experiment, the students were provided with informed consent.

### Data Analysis

To answer the first research question students’ written assignments were collected by the end of the intervention to be evaluated. A *t* test of students’ writing in both groups was done to compare students’ writing in the experimental and control groups. As shown in Table 1, students’ writing performance was analytically assessed in terms of language and reasoning abilities.

**Table 1:** Independent samples *t*-test comparing students learn CT skills utilizing Facebook (Intervention Group) and Control Group’ Improvements of Reasoning and Language Skills.

Variable	Control group			Intervention group			*t*	*p*
	*N*	*Mean*	*SD*	*N*	*Mean*	*SD*		
Reasoning	22	4.59	1.05	18	7.11	0.68	−8.77	0.000
Language	22	6.36	1.26	18	7.56	0.92	−3.35	0.000

## Results and Discussion

Students in the experimental group demonstrated significantly higher performance in the reasoning skills and writing skill:

1. There was a statistically significant difference in reasoning scores between the two groups of students, *t* (38) = −8.77, *p* < 0.00, two-tailed with the intervention group (*M* = 7.11, *SD* = 0.68) scoring higher than control group (*M* = 4.59, *SD* = 1.05). The magnitude of the differences in the means (mean difference = −2.52, 95% CI: −3.10 to −1.94) was large (eta squared = 0.67).
2. There was a statistically significant difference in language scores between the two groups of students, *t* (38) = −3.35, *p* < 0.00, two-tailed with the intervention group (*M* = 7.56, *SD* = 0.92) scoring higher than control group (*M* = 6.36, *SD* = 1.26). The magnitude of the differences in the means (mean difference = −1.19, 95% CI: −1.91 to −0.47) was large (eta squared = 0.23).

These significant differences are attributed to the reasoning thinking process that students in the experimental group applied. In fact, the infusion questions assisted students to deeply read the stories and represent their thoughts and comprehension of what they read in a skilled way through recognizing the reasoning elements of purpose, clarifying, questioning, summarizing, and connecting important ideas together. Overall, these students were more able to think deeper and write better, as they demonstrated the acquired ability to analyze the logic of the learning content, its purpose, its main questions, and the information it contains (Paul and Eldar, 2007).

The results support several studies which deal with the interrelation between critical thinking and writing improvement in various contexts (Yang et al., 2013; Harizaj and Hajrulla, 2017; Lu and Xie, 2019). Paul’s CT theory enhances students’ critical thinking and improves their writing skill and their writing organization in terms of rephrasing sentences, summarizing paragraphs, and identifying thesis statements, such improvements are mainly due to the practiced critical thinking skills (Afshar et al., 2017; Lu and Xie, 2019).

To answer the second research question “To what extent can Facebook as a learning platform stimulate students’ writing constructively?” Field notes were taken by the researcher during the 8 weeks intervention. They were mainly concerned with students’ interaction and participation on Facebook and compared with the other platforms (LMS) to explore the effectiveness of using Facebook as a constructivist tool for teaching and learning. For the experimental group, the researcher’s notes supported the suggestion of Mbati (2013) that critical discussions, whether live chat or posts and comments, proved to be ideal for utilizing constructivism in online education. Posting the set of critical thinking questions in the group encouraged constructivist learning as the students learned through collaboration and interaction with their peers. Their posts and comments indicated their improvement and transformation from passive to active learners capable of forming their subjective understanding and comprehension of the learning content and of analyzing it based on the critical thinking skills they had received.

The students interacted using Facebook on three levels: interaction with the learning content, with the instructor and with their peers. First, students’ interaction with the learning content was done by analyzing the short stories using the infused critical thinking skills. These skills enabled students to be the leaders of their learning; they had to come up with their own analyses and viewpoints. Although they had some critical thinking elements and standards to follow, but still it was their task to reach subjective conclusions. There was no one right answer and all the answers were assessed based on the critical thinking standards. Students’ responses to some questionnaire items such as “The course has helped me understand how to read literary works,” “I can better develop relevant ideas about the studying topics,” and “I can better use supporting information to express my viewpoints” with the majority agreed/strongly agreed showed that the critical thinking skills contributed to creating a thinking map for the students which leads to better learning.

**Figure 1:**
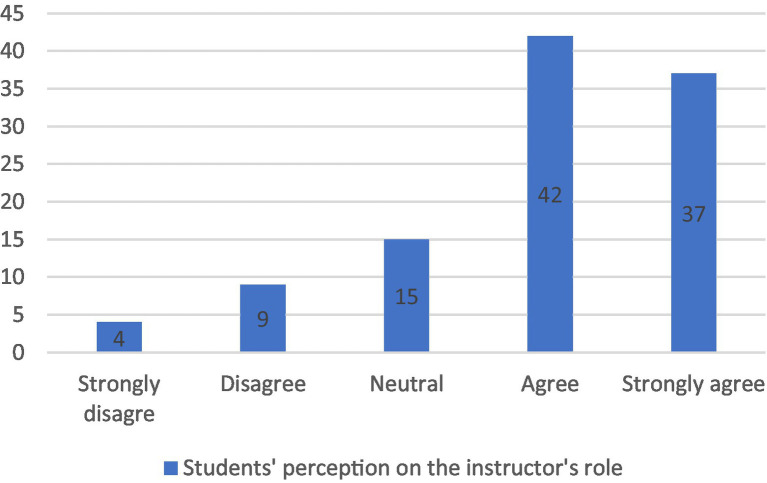
Students’ perception of the instructor’s role.

Second, students’ interaction with the instructor took on a new turn, i.e., the instructor’s role was no longer to lead the learning process, as the case with the control group, but to trigger students’ thinking and give them feedback where necessary. Her constructivist teaching role supports the argument of Li (2011), who emphasizes the role of teachers in finding a fruitful space for learning through thinking. Indeed finding such a space for all the students to participate, using Facebook facilities such as commenting and tagging, helped the instructor to determine their levels, their progress in content learning, and their perception of critical thinking in addition to their writing ability. Consequently, she was able to assess each student individually according to their output. Although all the experimental group students were involved in the discussions, the researcher could identify their different levels, abilities, and needs from their comments, and interaction with their peers, and she gave personalized feedback accordingly. This was reflected in the students’ attitude toward the teacher, as shown in Figure 1 below.

Students’ interaction with their peers facilitated collaborative and interactive learning. By commenting on their peers’ posts to ask for elaboration, agreeing, or disagreeing, giving examples or using other critical thinking standards, the students were able to interact more deeply and significantly. In addition, they acquired new vocabulary and grammar structures from one another. In several posts, the researcher found some students asking their peers about the meaning of some words or suggesting better words to express the same idea. These results support of Alotumi (2015) who investigated the effects of peer feedback on online learning groups and found out that learners became more active and confident, and improved their own writing by reading and commenting on their peer’s writing. Like the findings of other studies (Sirivedin et al., 2018; Hasan et al., 2019), the researcher also noticed how students improved their grammar and vocabulary and how they exchanged information and sought advice and clarification as part of their learning process. This informal way of learning significantly helped the students improve their writing skills and their critical thinking skills.

Involving students and making them responsible for their learning created a sense of enjoyment and commitment. For example, receiving notifications for new updates in the group, using emojis and tagging each other created a sense of friendly atmosphere, and relieved the students from the pressure of accomplishing the tasks in a short time. In fact, the majority of the students responded to the following questionnaire items “the course workload was appropriate for the course level” and “the amount of studying hours I needed at home to do required tasks was appropriate” with *agree* and *strongly agree*. No students *disagreed/strongly disagreed* with these statements, but there were a few neutral responses. This result is also articulated in studies of Wu (2016) and Aziz and Khatimah (2019) in which they agreed that utilizing Facebook has various advantages: the participants’ enjoyment of the online writing classes, openness, flexibility, accessibility, interactivity, and timeliness.

On the other hand, the researcher noticed that although the students in the control group had the chance to post their questions and inquiries during and after the lectures, their participation was very limited, and almost all of the questions were about the exams and marks. For example, once a student asked “if I memorize your presentations would I guarantee passing the course?” then a flow of questions started pouring regarding the materials they have to study (memorize) and the word count and the marks they would lose if they do not reach it. These questions showed that students were not really involved in the learning process; instead, how to pass the course. This attitude could be attributed to the instructional method of teaching which excluded students from creating knowledge and coming up with their own ideas; their post-test results confirmed their weaknesses in creating substantive pieces of writing, as the traditional curricula do not include the critical writing skills practiced in the intervention.

Further, comparing the longer lectures with less communication and interaction in the control group to the shorter live sessions in which all the students were involved in the experimental group, showed a significant difference between them. That also justifies the significant difference in the writing results, as Mayer and Wittrock (1996) claimed, communication played a vital role in enhancing the learning experience as students shared and exchanged their thoughts and information.

These critical thinking practices allowed students to learn out of the box and integrate their previous experiences and knowledge into the learning content, which is an important criterion for knowledge construction in the constructivist theory. Supporting the findings of other studies (Sirivedin et al., 2018; Hasan et al., 2019), the researcher’s notes revealed how students improved their grammar and vocabulary when learning English informally online as well as how they exchanged information and sought advice and clarification as part of their learning process, which significantly helped improving their writing skills in addition to their critical thinking skills.

The questionnaire was distributed to the experimental group students to explore their perception of this critical thinking-infused constructivist course model. Students’ responses in the questionnaire supported recommendations of Manca and Ranieri (2016) for utilizing Facebook for educational purpose. Facebook is found to contribute to create and develop new different roles for learners and teachers and facilitate applying new methods of communication and collaboration within educational contexts.

**Figure 2:**
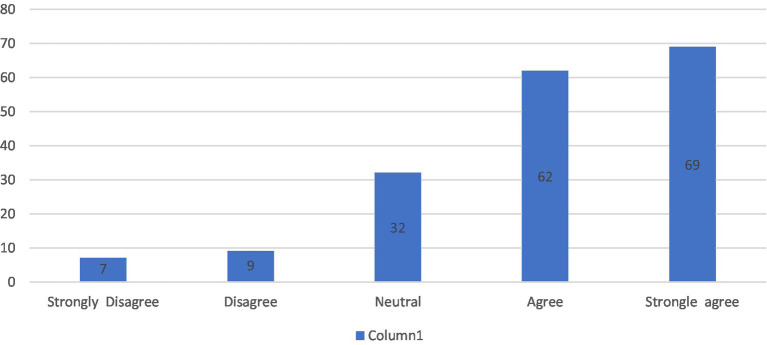
The overall impact of the intervention on the participants.

For the first section of the questionnaire “The overall impact of the intervention on the participants,” only 4%–5% of the students believed that the intervention did not have a positive impact on their learning, 18% percent of the students chose neutral, while 74% ranged between *agree* and *strongly agree*. Therefore, it can be concluded that the majority believed that the intervention introduced them to critical thinking, made them aware of its importance in learning and helped them better study literature courses. Also the workload and time allocated were appropriate (Figure 2).

**Figure 3:**
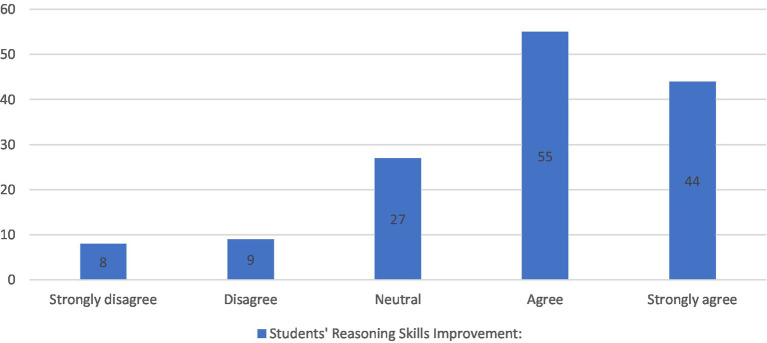
Students’ reasoning skills improvement.

**Figure 4:**
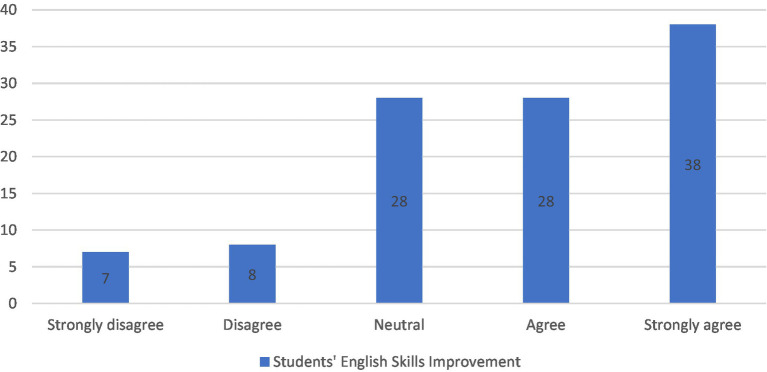
Students’ English skills improvement.

For the next two sections, “Students’ CT skills improvement” and “Students’ English skills improvement,” as shown in Figures 3, 4 respectively, the majority agreed that the intervention had significant impact on acquiring and improving their skills. About 69% of students *agreed* and *strongly agreed* that the intervention enhanced their critical thinking skills, namely understanding the importance of implementing reasoning skills in English courses to better analyze authors’ arguments and support with significant information and elaboration. In addition, they believed that they learned to differentiate between facts and assumptions, make clearer inferences and develop relevant ideas about the topics under study. As for the second section, 61% believed that their English improved alongside the learning content, as the intervention increased their confidence in using English language to express their thoughts and beliefs orally in live discussions or in a written form on the Facebook group. However, neutral answers in both sections were 19 and 26% and *disagree* responses were 12 and 13%, respectively. These results are most probably due to the time limitations of the study, as less able students needed more time to be able to use the critical thinking skills smoothly and be comfortable with the constructivist mode of learning. Nevertheless, their posttests showed higher grades than their peers in the control group.

**Figure 5:**
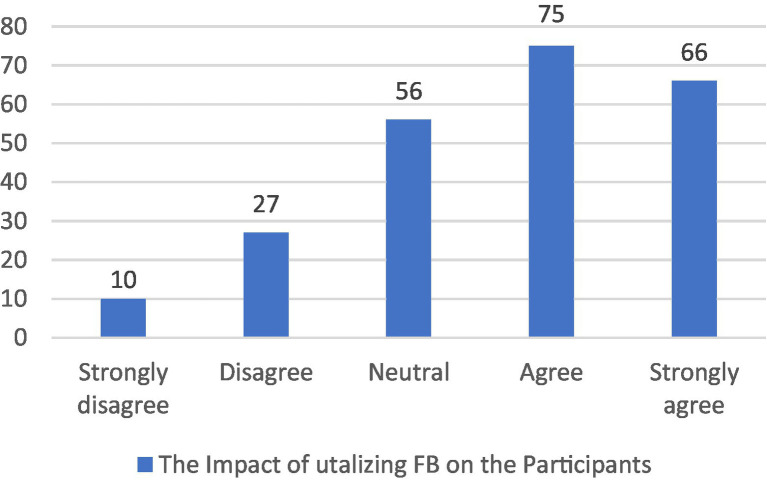
The impact of utilizing literature circles and socratic discussions on the participants.

For utilizing Facebook as a learning tool, the majority *agreed/strongly agreed* that Facebook facilitated their learning and their critical thinking enhancement and increased their communication and interaction as they cooperated and collaborated with their peers and the instructor, which also increased their motivation and involvement (Figure 5). On the other hand, 74% of the students found that the instructor was successful in redesigning the course for the sake of infusing the critical thinking skills, and she clearly introduced it to them. Also she effectively organized and facilitated the Facebook group and live chat discussions, challenged them to do their best and provided individualized feedback on their writing, an act that helped them recognize their weaknesses and work on them (Figure 1). The responses support findings of Schrader (2015) that the Facebook group contributes to finding a space to build shared meaning and personal connections between the students and their peers, their teacher, and the learning content.

The results of the questionnaire showed that the intervention which was built on the constructivist and infusion approaches for teaching literature is welcomed by students, as their responses were generally positive toward infusing critical thinking in the learning content for a more comprehensive and substantive use of language.

## Limitations

The results of the current study show significant differences between the experimental and control groups, and support results of several studies in the literature. However, the study still suffered from a few limitations. These include the short period of time, the small sample size, and disregard of students’ variables of gender. More studies have to be conducted in order to get more generalizable results.

## Conclusion

As mentioned earlier, this study did not aim to design a new course for teaching critical thinking but to redesign the teaching instruction through the infusion of a set of critical thinking skills in an English literature course, namely during COVID-19 pandemic, as mostly English language students in Saudi Arabia have been not very successful in improving their writing skills significantly prior the pandemic.

Using social media, namely Facebook, aimed to facilitate constructivist leaning and make the learning context friendly and easy to handle by all students through self, peers’, and instructor’s assessment. With the upcoming COVID-19 lockdown, educational sectors more than any time before have to put effective and workable alternatives for such exceptional circumstances. As many issues may confront students during their new online learning experience, such as motivation, self-confidence, anxiety, hesitation, and English language abilities; as teaching in class is different from teaching online from various perspectives and using same teaching techniques would merely lead to same outcomes, which were not expected. Believing that there should be well-designed teaching instructions and materials for effective education, this study aims to integrate social media, namely Facebook, as a means to overcome students’ hurdles and improve their critical thinking and English language skills during the outbreak, as it supports the claim of Paul and Elder (2001) that the new teaching methods and curricula have to keep critical thinking in mind as the quality of students’ thinking today determines the quality of the world they create tomorrow.

Finally, this study aims to draw educators and stakeholders’ awareness to their students’ obstacles improving their writing and provide a solution to their problems through suggesting a course design that addresses such obstacles and challenges and offers students a joyful and fruitful learning experience, so that no learners would be left behind in improving the target skills while studying online.

## Ethics Statement

The studies involving human participants were reviewed and approved by Near East University Ethical Committee Board. The patients/participants provided their written informed consent to participate in this study. Written informed consent was obtained from the individual(s) for the publication of any potentially identifiable images or data included in this article.

## Author Contributions

All authors listed have made a substantial, direct, and intellectual contribution to the work and approved it for publication.

## Conflict of Interest

The authors declare that the research was conducted in the absence of any commercial or financial relationships that could be construed as a potential conflict of interest.

## Publisher’s Note

All claims expressed in this article are solely those of the authors and do not necessarily represent those of their affiliated organizations, or those of the publisher, the editors and the reviewers. Any product that may be evaluated in this article, or claim that may be made by its manufacturer, is not guaranteed or endorsed by the publisher.
