# Supplementary material for: Critical Thinking for Writing Using Facebook Under COVID-19 Lockdown: A Course Model for English Literature Students
Source: Front Psychol. 2022 Jun 3;13:903452. doi: 10.3389/fpsyg.2022.903452 (PMC9204049; doi:10.3389/fpsyg.2022.903452)
Supplement: Supplementary file 1 [file Table_1.DOCX]

**Appendices**

**Appendix A Critical Thinking & Language Rubric**

| Grade | Reasoning skills | Language & Organization |
| --- | --- | --- |
| A  9–10 points | Excellent (highly skilled).  The answer is accurate, insightful, clearly and precisely stated, and well exemplified (when an example is relevant). | - Has an introduction defining plan of essay/ Body divided into several paragraphs/ Conclusion which directly relates arguments to topic  - Error-free grammar  - Wide range of specialized terminology  -Consistent in-text citation and form of referencing |
| B  7–8 points | Commendable (skilled).  The answer is well expressed, though with minor problems. It is basically correct and clear. Any misunderstanding is minor. | - First four criteria above maintained- Demonstrates extensive grammar control  - Terminology specialized but less varied.- Minor Inconsistency in in-text citation and referencing |
| C  5–6 points | Mixed level (beginning skills).  The answer is clear but not perfectly accurate. It is partially correct and partially incorrect. | - Introduction and/or conclusion short but still satisfactory. - Evidence of editing.- Less grammar control than above.- Good range of specialized terminology.- Inconsistent in in-text citation and referencing |
| D  3–4 points | Poor (minimally skilled).  The answer, though partially accurate and minimally clear, is significantly inaccurate or misleading. | - Introduction and/or conclusion short but acceptable. - no evidence of editing.- Few grammatical errors that impede communication.- Above average range of specialized terminology.- Slightly confused introduction and/or conclusion, but body still fair.- No evidence of editing.- Some error types that impede communication.- Fair range of specialized terminology.- Inaccurate in-text citation and referencing |
| F  0–2 points | Unacceptable (unskilled).  The answer is inaccurate and/or unclear. | - No introduction and /or no conclusion.- Body badly organized or irrelevant.- Poor grammar control (extremely limited range of grammar & register).- Limited or not specialized range of terminology. - No in-text citations and no referencing |

**Appendix B Students’ Perception of the Course Model Questionnaire**

| *1* | *The Overall Impact of the Course on the Participants* | *1 strongly disagree/ 5 strongly agree* |
| --- | --- | --- |
| 1.1 | The course has increased my critical thinking skills . | 1 2 3 4 5 |
| 1.2 | The course has increased my interest in critical thinking. | 1 2 3 4 5 |
| 1.3 | The thinking skills I have acquired in this course are important. | 1 2 3 4 5 |
| 1.4 | The course was well organized. | 1 2 3 4 5 |
| 1.5 | The course workload was appropriate for the course level. | 1 2 3 4 5 |
| 1.6 | The amount of studying hours I needed at home to do required tasks was appropriate. | 1 2 3 4 5 |
| 1.7 | The course has helped me understand how to read literary works. | 1 2 3 4 5 |
| 1.8 | Using modern English novels was appropriate to the goals of the course. | 1 2 3 4 5 |
| 1.9 | The course has given me confidence to use critical thinking skills in other subjects. | 1 2 3 4 5 |
| 1.10 | The course has increased my ability to relate what I studied at university to real life. | 1 2 3 4 5 |
| 1.11 | After I completed the course I began having a new outlook on life and current events. | 1 2 3 4 5 |
| *2* | *Students' Reasoning Skills Improvement* | *1 strongly disagree/ 5 strongly agree* |
| 2.1 | I understand the importance of implementing reasoning skills in learning English. | 1 2 3 4 5 |
| 2.2 | I can better analyze authors' arguments. | 1 2 3 4 5 |
| 2.3 | I can better use supporting information to express my viewpoints. | 1 2 3 4 5 |
| 2.4 | I can better elaborate on different viewpoints. | 1 2 3 4 5 |
| 2.5 | I can better differentiate between facts and assumptions. | 1 2 3 4 5 |
| 2.6 | I can better develop relevant ideas about the studying topics. | 1 2 3 4 5 |
| 2.7 | I can make clearer inferences when reading the studying materials. | 1 2 3 4 5 |
| 2.8 | I can answer complex questions more deeply. | 1 2 3 4 5 |
| *3* | *Students' English Skills Improvement* | *1 strongly disagree/ 5 strongly agree* |
| 3.1 | The course has helped me further develop my reading skill. | 1 2 3 4 5 |
| 3.2 | The course has helped me further develop my writing skill. | 1 2 3 4 5 |
| 3.3 | The course has increased my confidence in using English language to express my thoughts and beliefs. | 1 2 3 4 5 |
| *4* | *The Impact of Facebook on the Participants* | *1 strongly disagree/ 5 strongly agree* |
| 4.1 | By using Facebook I had more time to practice English than in traditional classes. | 1 2 3 4 5 |
| 4.2 | Facebook facilitated my learning. | 1 2 3 4 5 |
| 4.3 | Using Facebook encouraged me to express my ideas and thoughts. | 1 2 3 4 5 |
| 4.4 | Using Facebook increased my ability to work collaboratively with other students. | 1 2 3 4 5 |
| 4.5 | Facebook was an effective tool for enhancing my critical thinking. | 1 2 3 4 5 |
| 4.6 | I always learnt something new from the Facebook discussions. | 1 2 3 4 5 |
| *5* | *The Instructor* | *1 strongly disagree/ 5 strongly agree* |
| 5.1 | Prior to starting the course, the instructor focused my attention on the reasons for taking the course. | 1 2 3 4 5 |
| 5.2 | The instructor provided helpful feedback continuously. | 1 2 3 4 5 |
| 5.3 | The instructor provided a clear evaluation of my writing in the Facebook group. | 1 2 3 4 5 |
| 5.4 | The instructor effectively organized the Facebook group discussions. | 1 2 3 4 5 |
